# Adjunctive Autologous Platelet-Rich Plasma Activated with Autologous Thrombin Serum for a Refractory Below-Knee Amputation Stump Wound: A Case Report

**DOI:** 10.3390/reports9040351

**Published:** 2026-10-14

**Authors:** Khansa Shara, Hasan AlAjmi, Nada Baatiah, Sara Darag, Renad Bakhamis, Amr Abdelhakim

**Affiliations:** 1 Nursing Department, Johns Hopkins Aramco Healthcare, Dhahran 31311, Saudi Arabia; khansa.shara@jhah.com (K.S.); sara.darag@jhah.com (S.D.); 2 Surgery Department, Johns Hopkins Aramco Healthcare, Dhahran 31311, Saudi Arabia; hasan.alajmi@jhah.com (H.A.); amr.ahmed2@jhah.com (A.A.); 3 Research Office, Johns Hopkins Aramco Healthcare, Dhahran 31311, Saudi Arabia; 4 College of Medicine, Umm Al-Qura University, Makkah 21955, Saudi Arabia; renadbakhamis17@gmail.com

**Keywords:** chronic wound healing, platelet-rich plasma, amputation stump, below-knee amputation, case report

## Abstract

**Background and Clinical Significance:** Refractory below-knee amputation (BKA) stump wounds can delay prosthetic rehabilitation. This report describes wound healing during multimodal care that included autologous platelet-rich plasma (PRP) activated with autologous thrombin serum (ATS); **Case Presentation:** A 33-year-old man with Charcot–Marie–Tooth disease and a persistent right BKA stump wound after surgical revisions underwent three PRP–ATS applications on 16 and 23 February and 8 March 2026. Collagen dressings were applied on 2 and 15 March, and compression was continued thereafter. The estimated wound volume decreased from 14.18 cm^3^ at baseline to 0.48 cm^3^ at week 3 (approximately 97%); advanced epithelialization was observed at week 4. Complete closure was documented on 16 April 2026, approximately eight weeks after treatment began. No treatment-related adverse events were reported. After closure, the patient was referred for prosthetic rehabilitation assessment; fitting and functional use were not documented; **Conclusions:** Healing was temporally associated with PRP–ATS as part of a multimodal treatment course. This single case supports the feasibility of the approach but cannot establish the independent efficacy of PRP–ATS.

## 1. Introduction and Clinical Significance

Postoperative wound complications following lower-extremity amputation remain a significant clinical challenge, often delaying stump healing, prosthetic fitting, rehabilitation, and ultimately functional recovery [1]. Successful management of amputation stump wounds is essential not only to restore skin integrity but also to minimize morbidity, reduce healthcare utilization, and improve patients’ quality of life. However, a subset of patients develops chronic, non-healing wounds despite repeated surgical interventions and comprehensive wound care, creating a considerable therapeutic challenge.

Chronic wounds are defined as wounds that fail to progress through the normal phases of healing in an orderly and timely manner, typically persisting for more than three months [2]. Their pathophysiology is characterized by persistent inflammation, excessive proteolytic activity, degradation of essential growth factors, impaired angiogenesis, and extracellular matrix dysfunction, all of which disrupt normal tissue repair and regeneration [3]. These biological abnormalities often render conventional wound management strategies insufficient, highlighting the need for novel regenerative approaches.

Autologous platelet-rich plasma (PRP) has emerged as a promising biological therapy for chronic wound management because of its regenerative potential. Prepared from centrifuged autologous whole blood, PRP contains a concentrated population of platelets that release numerous bioactive growth factors upon activation, including platelet-derived growth factor (PDGF), transforming growth factor-beta (TGF-β), vascular endothelial growth factor (VEGF), and epidermal growth factor (EGF). These mediators may support different stages of wound healing by stimulating angiogenesis, fibroblast proliferation and migration, collagen synthesis, extracellular matrix deposition, granulation tissue formation, and keratinocyte migration and re-epithelialization. PRP may also modulate the inflammatory response and provide a provisional fibrin matrix that supports cellular migration and tissue organization [4,5]. However, PRP products are biologically heterogeneous because their platelet concentrations, leukocyte content, activation methods, preparation systems, and administration protocols vary considerably. Therefore, biological plausibility should not be interpreted as proof of clinical efficacy for every wound type [5,6].

The clinical efficacy of PRP has been increasingly demonstrated in chronic wounds, such as diabetic foot ulcers and chronic leg ulcers, with systematic reviews and meta-analyses reporting improved healing rates and shorter time to wound closure compared with conventional therapy [7,8]. Nevertheless, the certainty and generalizability of these findings are limited by variations in wound etiology, PRP preparation and activation methods, treatment frequency, outcome definitions, comparators, follow-up periods, and concomitant wound-care interventions. Evidence specifically addressing refractory postoperative amputation stump wounds remains scarce. Therefore, findings from other chronic wound populations cannot be directly extrapolated to this clinical setting.

Beyond wound management, PRP has been investigated as an autologous biological treatment across regenerative and musculoskeletal medicine. Its applications include tendinopathies, muscle and ligament injuries, osteoarthritis, cartilage disorders, and other musculoskeletal conditions. For example, PRP, alone or combined with hyaluronic acid, has been evaluated as an intra-articular treatment for knee osteoarthritis [9].

Autologous thrombin serum (ATS), obtained from the patient’s own blood, can be used as a biological activator of PRP. When combined with PRP, ATS promotes the conversion of fibrinogen into fibrin, producing a platelet-containing gel that acts as a temporary biological scaffold. This fibrin matrix may enhance the local retention of platelets and their bioactive mediators while supporting cellular migration, angiogenesis, extracellular matrix deposition, and tissue repair. Because ATS is autologous, it may reduce the immunological and infectious risks associated with exogenous thrombin products [10,11].

This case report describes the successful use of adjunctive autologous PRP in the treatment of a refractory below-knee amputation stump wound that failed to heal despite repeated surgical debridement, wound revision, and standard wound care. The report highlights the potential role of PRP as a regenerative adjunct to promote wound closure and facilitate progression to prosthetic rehabilitation in patients with complex postoperative stump wounds.

## 2. Case Presentation

### 2.1. Patient Information

A 33-year-old man (body mass index, 35.38 kg/m^2^) had established hereditary motor sensory neuropathy (Charcot–Marie–Tooth disease), with recurrent bilateral neuropathic ulcers since childhood, Charcot arthropathy, chronic infections, and multifocal osteomyelitis. Available records did not document additional neurological, genetic, metabolic, immunological, or vascular investigations during the present treatment episode.

He underwent left BKA on 12 March 2015, with satisfactory recovery, and right BKA on 25 September 2024 for neuropathic destruction and osteomyelitis. The right stump subsequently dehisced. Irrigation, debridement, and closure were performed on 31 July 2025; excision of the chronic wound, debridement, and primary closure followed on 29 December 2025. The wound remained open despite surgical follow-up and multiple local dressings (Table 1).

He had no history of diabetes mellitus, hypertension, or other major systemic comorbidity, no known drug allergies, and no routine long-term medication. Histopathology of the amputated right limb showed ulceration with granulation and necrotic tissue consistent with gangrene, underlying atherosclerotic vascular narrowing, and viable soft-tissue resection margins.

### 2.2. Clinical Findings and Diagnostic Assessment

After the December 2025 revision, a superficial area of dehiscence persisted despite Inadine and subsequent honey-based dressings. At baseline on 16 February 2026, the central wound measured 1.5 × 3.5 × 2.7 cm, with an estimated volume of 14.18 cm^3^; the wound bed comprised 95% granulation tissue and 5% slough. Moderate serous exudate and stump edema were present, and the periwound skin was intact.

No purulence, surrounding cellulitis, fluctuance, or other clinical signs of acute stump infection were documented before PRP treatment. A recorded white blood cell count was 7.7 × 10^9^/L. No cultures, erythrocyte sedimentation rate, C-reactive protein measurement, advanced imaging, or targeted investigation for residual osteomyelitis was obtained before PRP; active infection therefore was not excluded by microbiological or imaging assessment.

Stump perfusion was evaluated clinically at wound-care and surgical visits, without clinical evidence of critical limb ischemia. Objective perfusion testing or formal vascular consultation was not performed during the PRP treatment period, despite the vascular narrowing reported in the amputated specimen.

#### Diagnostic Challenges

The established neuropathy, prior osteomyelitis, repeated wound dehiscence, and histological vascular narrowing could each have affected healing. Without objective perfusion testing or a focused evaluation for residual osteomyelitis, their relative contributions cannot be determined.

### 2.3. Therapeutic Interventions

After prolonged local wound care and two revision procedures, PRP activated with ATS was introduced as one component of the wound-care regimen. Three applications were performed on 16 February, 23 February, and 8 March 2026; collagen dressings were used between and after these applications (Table 1 and Table 2).

#### PRP Preparation and Application Protocol

At each PRP–ATS session, 20 mL of peripheral venous blood was collected into two 10 mL Regen BCT tubes and centrifuged at 1500× *g* for 5 min using the RegenPRP™ system (Regen Lab SA, Le Mont-sur-Lausanne, Switzerland). Approximately 5 mL of PRP was recovered from each tube, yielding approximately 10 mL in total. A separate 10 mL of whole blood was collected into a dedicated RegenATS tube containing separator gel and centrifuged immediately, without a waiting period before centrifugation, at 1500× *g* for 5 min. Approximately 2 mL of ATS was recovered. The presence or type of clot activator in the tube, and the elapsed time from preparation to application, were not documented in the available treatment record.

The approximately 10 mL of PRP and 2 mL of ATS were mixed directly at a 5:1 volume ratio according to the manufacturer’s protocol and allowed to interact for approximately 3 min to form a platelet gel before placement into the wound cavity. Gel formation was observed clinically; no quantitative gelation assessment was performed. No calcium chloride or exogenous thrombin was added. According to manufacturer-provided data, this system is expected to yield leukocyte-poor PRP with greater than 80% platelet recovery and approximately 96% granulocyte depletion. Baseline platelet count, final platelet and leukocyte concentrations, and ATS thrombin concentration or activity were not measured in this patient.

At each application, approximately 12 mL of the activated PRP mixture was placed into the wound cavity and covered with an occlusive dressing and a compression wrap for approximately 7 days. Wounds were cleansed with normal saline before dressing changes. On 2 March, a Pro-Heal collagen dressing with a semi-occlusive cover and compression was used for approximately five days; on 15 March, collagen, an occlusive cover, and compression were applied. Collagen dressings and compression continued until closure. Compression was used to manage edema and stabilize the dressing; the compression pressure, exact cover-dressing materials, and subsequent change frequency were not recorded. Pressure and friction on the stump were avoided. No sharp debridement was needed during the PRP treatment period, and no systemic therapy specific to this treatment episode was documented in the supplied clinical chronology.

**Figure 1:**
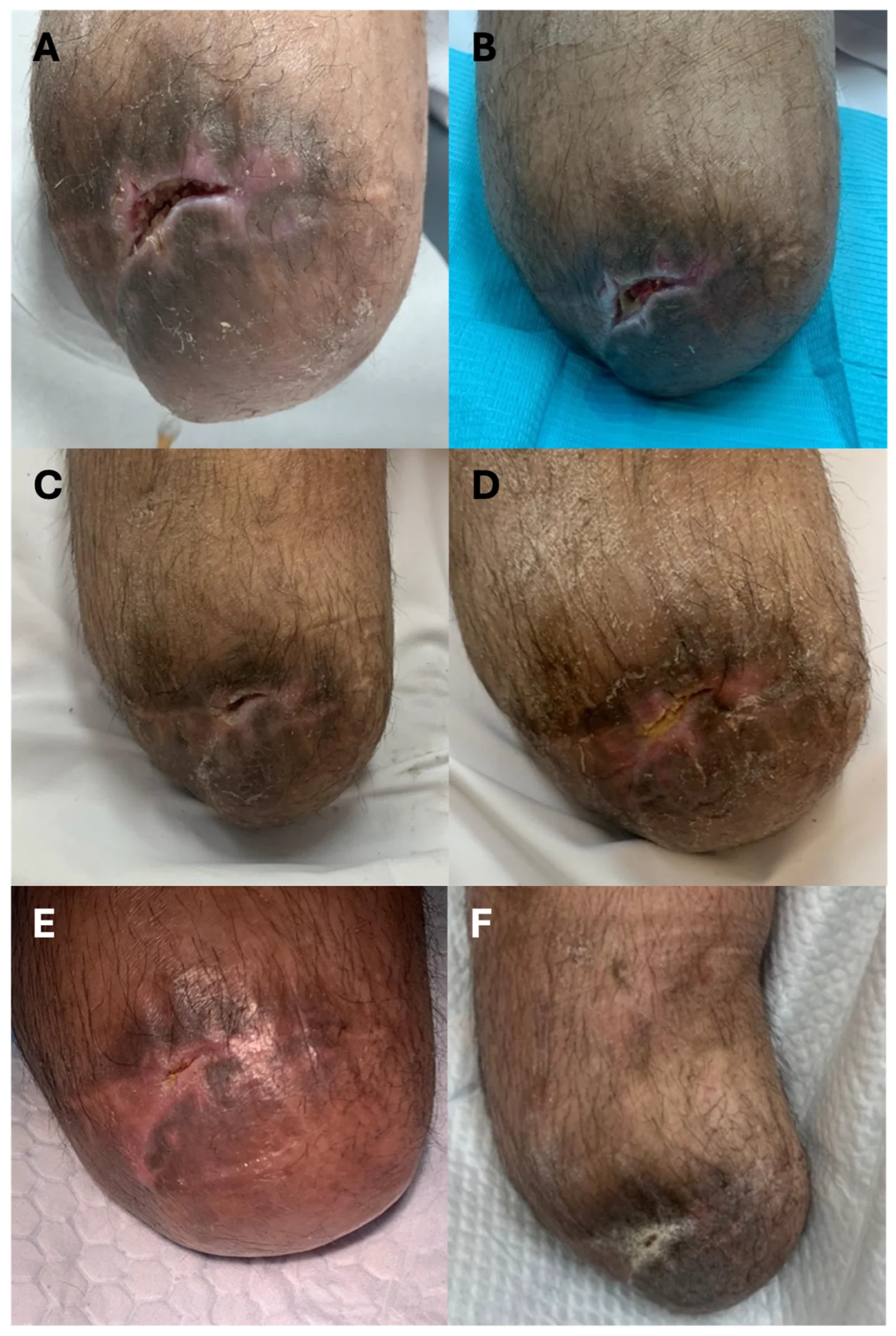
Serial clinical photographs of the right BKA stump wound: (**A**) 16 February 2026, baseline; (**B**) 23 February, week 1; (**C**) 2 March, week 2; (**D**) 8 March, week 3; (**E**) 15 March, week 4; and (**F**) 16 April, documented closure. Photographs were obtained during routine care without uniform positioning or a calibrated scale across all panels.

The wound-care service recorded maximum length, width, and depth with a standard assessment ruler at serial visits; the same team assessed the wound whenever possible. The estimated volume was calculated as length × width × depth. This rectangular estimate may differ from the true volume of an irregular wound. Photographs were taken during routine care without consistent camera distance, angle, lighting, orientation, or a calibrated scale in every image (Figure 1).

### 2.4. Follow-Up and Outcomes

No treatment-related adverse events were reported during the three PRP–ATS applications. No additional surgery was performed during this treatment course.

The estimated volume decreased from 14.18 cm^3^ at baseline to 0.48 cm^3^ on 8 March 2026, an approximately 97% reduction after three weeks. The wound showed complete granulation by 2 March and advanced epithelialization by 15 March; complete closure had not yet been documented at week 4 (Figure 1).

Complete epithelialization and wound closure were documented on 16 April 2026, approximately eight weeks after the first PRP–ATS application. After closure, the patient was referred for prosthetic rehabilitation assessment. Final fitting, prosthesis use, loading tolerance, mobility, recurrent breakdown, and later follow-up were not available.

This case report was prepared in accordance with the CARE reporting guidelines. The completed CARE checklist is provided as Supplementary Table S1.

## 3. Discussion

Persistent wound breakdown following below-knee amputation represents a challenging postoperative complication that can delay prosthetic fitting, prolong rehabilitation, increase healthcare utilization, and adversely affect functional recovery. Management becomes particularly difficult when wound healing fails despite repeated surgical revision and conventional wound care [1]. In the present case, wound closure was documented during a treatment course that included three PRP–ATS applications after earlier surgical revisions.

The favorable clinical outcome observed in this patient is biologically plausible given the proposed actions of platelet-derived mediators. Chronic wounds are characterized by persistent inflammation, impaired angiogenesis, degradation of endogenous growth factors, and extracellular matrix dysfunction, resulting in failure to progress through the normal phases of wound healing [3]. PRP provides a concentrated source of platelets that release multiple bioactive mediators, including platelet-derived growth factor (PDGF), transforming growth factor-beta (TGF-β), vascular endothelial growth factor (VEGF), and epidermal growth factor (EGF), all of which promote angiogenesis, fibroblast proliferation, extracellular matrix synthesis, epithelialization, and modulation of the inflammatory response [12,13]. These proposed mechanisms could be relevant to the observed granulation and wound closure, although they were not measured in this patient.

ATS was used to activate PRP and form a fibrin-containing gel prior to application, thereby potentially retaining the preparation locally. These are plausible mechanisms, but neither platelet concentration nor ATS thrombin activity was measured in this patient; therefore, the biological properties of this particular preparation cannot be quantitatively inferred.

Evidence from other chronic wound settings provides context for this case; systematic reviews and meta-analyses have demonstrated that PRP improves wound healing rates and increases the likelihood of complete wound closure compared with standard wound care in patients with chronic wounds, particularly diabetic foot ulcers [7,14,15]. Likewise, Tabanjeh et al. [16] reported favorable outcomes following autologous PRP therapy in patients with chronic lower-extremity ulcers.

Differences in wound cause, preparation, activation, application frequency, comparators, and accompanying care limit transfer of those findings to postoperative amputation stump wounds. The evidence for this specific indication remains sparse. Direct evidence for PRP–ATS in this setting remains insufficient to infer benefit from studies of other wound etiologies.

The patient had undergone surgical revision prior to PRP–ATS, and compression and collagen dressings were used during subsequent healing. These measures, along with time and routine wound care, may have contributed. These observations document healing during a care plan that included PRP–ATS; they do not establish efficacy or identify which component contributed to closure.

Serial wound measurements and dated clinical photographs document the course. Limitations include a single patient, concurrent treatments, approximate volume estimates, nonstandardized photography, no objective perfusion testing or focused assessment for residual osteomyelitis, no patient-specific PRP cell counts or ATS thrombin assay, incomplete dressing specifications, and no documented prosthetic function or long-term outcome. The patient’s perspective was unavailable.

Evidence specific to postoperative amputation stump wounds, particularly with PRP–ATS, remains sparse; findings from other wound types cannot establish benefit in this setting.

## 4. Conclusions

Complete closure of a refractory right BKA stump wound was documented approximately eight weeks after the start of a multimodal course that included three PRP–ATS applications, collagen dressings, compression, and routine care. This case demonstrates feasibility and a temporal association with healing; it cannot establish the independent efficacy of PRP–ATS. Prosthetic use and longer-term durability remain unreported.

## Figures and Tables

**Table 1:** Clinical chronology before and during the PRP–ATS treatment course.

Date	Clinical Course	Management
Childhood–2015	Hereditary motor sensory neuropathy, recurrent neuropathic ulcers and osteomyelitis; left BKA on 12 March 2015	Prior wound care and debridements
25 September 2024	Right BKA for neuropathic destruction and osteomyelitis	Postoperative stump care
31 July–20 October 2025	Right stump dehiscence revised on 31 July; persistent gapping by October	Irrigation, debridement and closure; local dressings
November–December 2025	Persistent chronic wound; necrotic ulcer with rolled edges at 18 December review	Cavilon, Neogel and Aquacel Ag; excision, debridement and primary closure on 29 December
January–15 February 2026	Residual superficial dehiscence despite revision	Inadine, then honey-based dressings; PRP decision on 15 February
16 and 23 February 2026	Baseline and week 1 wound reviews	First and second PRP–ATS applications; occlusive cover and compression
2 and 8 March 2026	Week 2 fully granulating; week 3 volume 0.48 cm^3^	Collagen on 2 March; third PRP–ATS application on 8 March
15 March–16 April 2026	Week 4 advanced epithelialization; closure documented 16 April	Continued collagen dressings and compression; referred for prosthetic assessment after closure

**Table 2:** Wound measurements and treatment during multimodal care. Volumes are approximate rectangular estimates (length × width × depth).

Date	Wound Dimensions (L × W × D)	Wound Bed Composition	Exudate & Limb Status	Applied Dressing & Protocol
16 February 2026 (baseline)	1.5 × 3.5 × 2.7 cm (14.18 cm^3^)	95% granulation; 5% slough	Moderate serous exudate; edematous stump	PRP + ATS; occlusive cover and compression (~7 days)
23 February 2026 (week 1)	1.0 × 3.0 × 1.5 cm (4.50 cm^3^)	95% granulation; 5% slough	Mild serous exudate; decreasing edema	PRP + ATS; occlusive cover and compression (~7 days)
2 March 2026 (week 2)	1.0 × 2.0 × 0.5 cm (1.00 cm^3^)	100% granulation	Mild serous exudate; edema resolved	Pro-Heal collagen; semi-occlusive cover and compression (~5 days)
8 March 2026 (week 3)	0.8 × 2.0 × 0.3 cm (0.48 cm^3^)	100% granulation	Mild serous exudate; healthy periwound	PRP + ATS; occlusive cover and compression (~7 days)
15 March 2026 (week 4)	0.3 × 2.0 × 0.1 cm * (0.06 cm^3^ *)	Advanced epithelialization	Minimal serous exudate	Pro-Heal collagen; occlusive cover and compression
16 April 2026 (follow-up)	Complete closure	Complete epithelialization	No exudate	Collagen and compression continued until closure; no active dressing at follow-up

* The week 4 depth of 0.1 cm was an assumed value in the retrospective summary, rather than a confirmed measurement; the calculated volume of 0.06 cm^3^ is therefore provisional.

## Data Availability

The original contributions presented in this study are included in the article/Supplementary Material. Further inquiries can be directed to the corresponding author.

## Supplementary Materials

The following supporting information can be downloaded at: https://www.mdpi.com/article/10.3390/reports9040351/s1, Supplementary Table S1: CARE Checklist of Information to Include When Writing a Case Report.

## Abbreviations

The following abbreviations are used in this manuscript:

PRP	Platelet-Rich Plasma
ATS	Autologous Thrombin Serum
BKA	Below-Knee Amputation
PDGF	Platelet-Derived Growth Factor
TGF-β	Transforming Growth Factor-Beta
VEGF	Vascular Endothelial Growth Factor
EGF	Epidermal Growth Factor
