# Supplementary material for: Adjunctive Autologous Platelet-Rich Plasma Activated with Autologous Thrombin Serum for a Refractory Below-Knee Amputation Stump Wound: A Case Report
Source: Reports (MDPI). 2026 Oct 14;9(4):351. doi: 10.3390/reports9040351 (PMC13692453; doi:10.3390/reports9040351)
Supplement: Supplementary file 1 [file reports-09-00351-s001.zip › reports-4537567-supplementary.pdf]

CARE
case report guidelines

Supplementary Table S1:

CARE Checklist of information to include when writing a case report

(cc) BY-NC-ND

Topic	Item	Checklist item description	Reported on Line
Title	1	The diagnosis or intervention of primary focus followed by the words "case report"	Lines 2-4
Key Words	2	2 to 5 key words that identify diagnoses or interventions in this case report, including "case report"	Not reported
Abstract (no references)	3a	Introduction: What is unique about this case and what does it add to the scientific literature?	Lines 14-19
	3b	Main symptoms and/or important clinical findings	Lines 20-24
	3c	The main diagnoses, therapeutic interventions, and outcomes	Lines 24-30
	3d	Conclusion—What is the main "take-away" lesson(s) from this case?	Lines 31-34
Introduction	4	One or two paragraphs summarizing why this case is unique (**may include references**)	Lines 37-72
Patient Information	5a	De-identified patient specific information.	Lines 75-93
	5b	Primary concerns and symptoms of the patient.	Lines 75-88
	5c	Medical, family, and psycho-social history including relevant genetic information	Partial: FH/psychosocial NR
	5d	Relevant past interventions with outcomes	Lines 81-99
Clinical Findings	6	Describe significant physical examination (PE) and important clinical findings.	Lines 100-107
Timeline	7	Historical and current information from this episode of care organized as a timeline	Table 1; lines 142-154
Diagnostic Assessment	8a	Diagnostic testing (such as PE, laboratory testing, imaging, surveys).	Lines 89-107
	8b	Diagnostic challenges (such as access to testing, financial, or cultural)	Lines 108-115
	8c	Diagnosis (including other diagnoses considered)	Lines 75-115
	8d	Prognosis (such as staging in oncology) where applicable	N/A
Therapeutic Intervention	9a	Types of therapeutic intervention (such as pharmacologic, surgical, preventive, self-care)	Lines 116-140
	9b	Administration of therapeutic intervention (such as dosage, strength, duration)	Lines 120-140
	9c	Changes in therapeutic intervention (with rationale)	Table 1; lines 142-145
Follow-up and Outcomes	10a	Clinician and patient-assessed outcomes (if available)	Lines 155-169
	10b	Important follow-up diagnostic and other test results	Table 1; lines 159-169
	10c	Intervention adherence and tolerability (How was this assessed?)	Lines 155-158
	10d	Adverse and unanticipated events	Lines 155-158
Discussion	11a	A scientific discussion of the strengths AND limitations associated with this case report	Lines 212-240
	11b	Discussion of the relevant medical literature **with references**.	Lines 192-204
	11c	The scientific rationale for any conclusions (including assessment of possible causes)	Lines 180-211
	11d	The primary "take-away" lessons of this case report (without references) in a one paragraph conclusion	Lines 243-249
Patient Perspective	12	The patient should share their perspective in one to two paragraphs on the treatment(s) they received	Not reported
Informed Consent	13	Did the patient give informed consent? Please provide if requested	Yes ☑ No ☐
